# Supplementary material for: Multigene typing of Giardia Duodenalis isolated from tuberculosis and non-tuberculosis subjects
Source: PLoS One. 2023 Mar 23;18(3):e0283515. doi: 10.1371/journal.pone.0283515 (PMC10035859; doi:10.1371/journal.pone.0283515)
Supplement: Attachment — Submitted filename: Response to Reviewer.docx [file pone.0283515.s001.docx]

**Dear Dr.** **Hosseini Farash**

**Academic Editor**

**PLOS ONE**

**Manuscript ID: PONE-D-22-33229**

Thank you for allowing me to submit a revised draft of our manuscript titled: “**Multigene Typing of *Giardia Duodenalis* Isolated from Tuberculosis and Non-Tuberculosis Subjects”**. We appreciate the time and effort you have dedicated to providing your valuable feedback on our manuscript. We are grateful to the reviewers for their insightful comments on our paper. We have been able to incorporate changes to reflect most of the suggestions provided by the reviewers. We have highlighted in yellow the changes within the manuscript.

Review Comments to the Author

Reviewer #1:

Response: We would like to thank reviewer for deep review and valuable comments and revisions. We hope this version could be suitable for publication.

1. Sample size is not sufficient for statistical analysis. increase the sample size of your study.

Response: With respect to reviewer, for this study we analyzed 30 Giardia-positive samples which were isolated from 427 samples during 2 years. We understand your concern, but there is no possibility to extend the number of samples on TB patients at this time.

2. Results reported in this study is different from authors' previous study (Ref. 19) including sample size and frequency of Giardia lamblia. The number of microscopically positive-Giardia samples in this study was reported 30, while in the previous study 8 samples were reported. Also the sample size of non-TB group is not the same as the previous study.

Response: Thank you so much for your valuable note. We sorry for this mistake. The number of samples was 427 not 327. Actually, sampling from TB patients was continued after our previous study (ref.19) to 261 TB samples (at the time of our previous study the number of samples was 161). In addition, in our previous study we just reported “Giardia-positive” for those samples which were definitively microscopically identical, but not for those that were suspected or have changes in their shapes (in this study we extracted DNA from both definitive positive and suspected samples). Therefore, the number of positive samples with molecular methods in 261 TB patients (considering all definitive and suspected Giardia positive by microscopy) was 13.

For healthy subjects, we have not employed positive samples from those healthy subjects which were included in our previous study. Giardia positive samples from non-TB patients was isolated from those apparently healthy subjects who referred to the Foodborne and waterborne Diseases Research Center at the same time.

Reviewer #2:

The paper is of interest in the field as scare data are available targeting Giardia coinfection with TB
The work was performed following the standard’s molecular approaches

Response: We would like to thank reviewer for deep review, valuable comments and revisions, and positive feedback. We hope this version could be suitable for publication.

Below few comments to improve the MS quality:

-English should be revised to avoid typo errors: e.g., L32, L41, L50, L57, L58, L61, L72, L131, L151, L186, L197, L209, L223, L231, L242...

Response: The manuscript has been reviewed again to improve English writing.

-In methodology part, authors should mention the multiple alignment software used in their analysis

Response: Thanks for comment. Relevant software (BioEdit) was added.

-In methodology part, authors should explain why they chose the "Tamura 3-parameter model" in their phylogenetic analysis

Response: Actually, "Tamura 3-parameter model" is this that “Tamura 3-parameter is the analyzing both transitional and transversional rates, G+C content bias, and correcting multiple hits”. This statement has been added to the relevant position in the methodology.

-Legends of Tables 2, 3 and 4 are misplaced

Response: Modification done!

-A footnote for Table 2 is required to understand the reported data

Response: Footnotes have been added for tables.

-Higher resolution of the figure needs to be provided

Response: Modification done!

Sincerely yours,

Dr. Hamed Mirjalali

Corresponding author
